# National availability, prices and affordability of essential medicines: a comprehensive analysis using WHO/HAI methodology in Chad

**DOI:** 10.11604/pamj.2025.51.72.47698

**Published:** 2025-07-09

**Authors:** Frank Edgard Zongo Ragomzingba, Saker Haoua Haroun, Colette Ngabéré, Ahmat Borgnol, Martine Yoyammel, Abatcha Oumar Kadai, John Eyong Efobi, Jacques Lukenze Tamuzi, Patrick de Marie Chimusa Katoto, Charles Shey Wiysonge, Blanche-Philomene Melanga Anya, Luc Zongo

**Affiliations:** 1 World Health Organization, Ndjamena, Chad,; 2 General Directorate of Pharmacy, Medicines and Laboratories, Ministry of Public Health, Ndjamena, Chad,; 3 Division of Epidemiology and Biostatistics, Department of Global Health, Faculty of Medicine and Health Sciences, Stellenbosch University, Cape Town, South Africa,; 4 Office of the President and CEO, South African Medical Research Council, Cape Town, South Africa,; 5 Centre for Tropical Diseases and Global Health, Department of Medicine, Catholic University of Bukavu, Bukavu, Democratic Republic of the Congo,; 6 Cochrane South Africa, South African Medical Research Council, Cape Town, South Africa,; 7 World Health Organization Regional Office for Africa, Brazzaville, Congo,; 8 Saint Thomas Aquinas Catholic University, Ouagadougou, Burkina Faso

**Keywords:** Essential medicines, availability, affordability, pricing, Chad

## Abstract

**Introduction:**

in Chad, the pharmaceutical sector faces many challenges, including essential medicine availability, affordability, and pricing. The country has a weak health system with limited access to medical care for the population. This study aims to assess the availability, affordability, and pricing of essential medicines in Chad from 1^st^ June to 31^th^ July 2023.

**Methods:**

we conducted a descriptive and cross-sectional study including public, private, and contractual sectors. Availability of essential medicines was measured as a percentage (%) of the surveyed outlets, and pricing was computed using the median price ratio (MPR).

**Results:**

our results showed that the average medicine availability ranged between 39.59% to 67.37%. In the public sector, average availability ranges from roughly 46% at the health center level to 61.4% at the provincial pharmacy level, with a 52.6% average across all health institutions. In the private sector, it is around 38% for private wholesalers, 48% for pharmacies, 58.4% for pharmaceutical depots, and 48% contractual sector. Provinces with the lowest availability rates were Batha, Kanem, Lac, and Hadjer Lamis, with percentages ranging between 39 and 43%. In terms of cost, the lowest priced generics (LGPs) are 9 times less than originator pharmaceuticals in the contracted sector, 4 times less in the public sector, and 2 times less in the private sector.

**Conclusion:**

this study's findings highlight the critical need to provide access to essential medicines and reduce their costs in Chad. Efforts should be undertaken to implement World Health Organization (WHO) guidelines for universal access to essential medicines in Chad.

## Introduction

Access to essential medicines is a vital component of the fulfilment of the right to the highest attainable standard of health [1,2]. As one of the Sustainable Development Goals (SDGs) of the United Nations, access to safe, effective, quality, and affordable essential medicines is important to health coverage for children by 2030 [1,3]. The recognition of the importance of essential medicines is not new. At the 1985 Nairobi Conference on the Rational Use of Drugs, government representatives and other stakeholders proposed a comprehensive set of essential medicines policies [4]. The Commission identified five areas that are crucial to essential medicines policies: paying for a basket of essential medicines, making essential medicines affordable, assuring the quality and safety of medicines, promoting the quality use of medicines, and developing missing essential medicines [4]. The pharmaceutical sector plays a crucial role in global public health, providing essential medicines for the treatment of diseases and prevention of infections. According to the WHO, access to medicines is a fundamental right and a key component of the right to health [5]. However, the accessibility and cost of essential medicines continue to pose significant challenges, especially in low- and middle-income countries (LMICs). Specifically, healthcare access in sub-Saharan Africa (SSA) was recorded at 42.56%, a notably low figure, despite SDG 3.8 aiming for universal health coverage to ensure that all individuals can obtain necessary health services [6].

In Chad, the pharmaceutical sector faces many challenges, including essential medicine availability and affordability. The country has a weak health system with limited access to medical care for the population. These statistics highlight the importance of ensuring the availability of essential medicines, which are crucial to reducing these rates and improving the overall health of the population. Elevated prices, combined with diminished purchasing power, restrict the financial accessibility of most individuals to pharmaceuticals. Analyzing the expenses of conventional therapies against the minimal daily salary in the public sector illustrates the financial strain these expenditures impose on individuals with low incomes in Chad. This prompts several patients to resort to the underground market for acquiring their medications. Numerous medications exhibit fluctuating prices throughout the various locations examined in the three sectors, including public, private, and contractual sectors; consequently, some individuals incur significantly higher costs for their treatments compared to alternative pharmaceutical stores. Numerous important medications were inadequately accessible in the public sector, whereas the same original drugs in the private sector were priced almost six times higher [7]. According to the World Bank, approximately 42% of the population lives below the poverty line [6]. Also, one of the main challenges is the inadequacy of health insurance mechanisms, which limit financial access to essential medicines for a large part of the population. According to a study conducted by the World Bank, less than 10% of Chadians have adequate health coverage [8]. This could be explained by inequalities in the distribution of medicines and their high cost are exacerbated by various factors, such as poverty, conflict, and insufficient health infrastructure. This lack of financial protection leads to catastrophic health expenditures for households, pushing many families into poverty and reducing their ability to afford medicines.

The last assessment of the availability and price of essential medicines took place in 2004 [9]. This survey will also allow us to assess the evolution of the therapeutic offer in the country since 2004. In this context, this study aims to assess the availability and price of essential medicines in Chad. The objective is to analyze the availability of essential medicines in private pharmaceutical establishments and health establishments, and assess the prices charged for these medicines. Specifically, this will involve (i) determining the availability of essential medical products in the different pharmaceutical sectors (public, contracted and private); (ii) determining the price that patients pay for essential medical products; (iii) determine the price difference between each originator drug and its cheapest generic equivalent; (iv) determine the price and availability ratios of medical products in the different private and public sub-sectors, and (v) compare pricing in Chad compared to other sub-Saharan African countries.

## Methods

We conducted a descriptive and cross-sectional study of the availability, prices, and affordability of children's essential medicines in Chad, using a standardized methodology developed by WHO and Health Action International (WHO/HAI) [10]. All data on the availability and prices of essential medicines in the public, private, and contractual sectors were collected from 1^st^ June to 31^st^ July 2023.

**Study settings:** the majority of the population in Chad lives below the poverty line, estimated at US$1 per day (4). Poverty affects more than half of Chadians, with 60% living in rural regions and 29% in cities. Unemployment affects the working population, especially in rural areas. This means that even when medicines are available, their cost can remain prohibitive for many Chadians. Access to affordable treatments is therefore a crucial issue for public health in Chad. The public sector enjoys competitive procurement rates; yet, medicines costs incurred by patients remain elevated [11]. An investigation of pricing disparities among health facilities based on necessary pharmaceutical costs shows that the price harmonization directive is not being properly implemented. Inadequate control and resources for pharmaceutical inspection contribute to this issue. The limited supply of generic pharmaceuticals in the private sector results in exorbitant prices in retail outlets. The prices of brand-name drugs are expensive and misaligned with the purchasing power of most folks [11]. Unregulated drug costs in Chad do not promote competition and so do not lower prices. Medicine prices have been marginally decreased in the religious sector. Religious health institutes purchase their goods from the Pharmaceutical Purchasing Centre (CPA) or import them directly [11].

The pharmaceutical sector has (i) a regulatory structure, which is the General Directorate of Pharmacy, Medicines and Laboratories (DGPML) which plays the role of National Pharmaceutical Regulatory Authority (ANRP); with a national drug commission in charge of approval, a committee of pharmacovigilance experts, (ii) supply and distribution structures. At the public level, the supply structures are the CPA and 23 Provincial Supply Pharmacies (PPA), which are currently autonomous and attached to the country's 23 provincial health delegations. At the private level, the latest mapping of establishments carried out in 2023 indicates 50 pharmacies, including 47 in Ndjamena, 205 pharmaceutical depots, including 147 in Ndjamena, and 60 pharmaceutical wholesalers, including 57 in Ndjamena. Among these private wholesalers, fewer than ten are operational and active. The country does not have a manufacturer. We also note the existence of a Logistics Management Unit (LMU) in charge of monitoring the availability and collection of logistics data and a national supply coordination commission with quantification subcommittees. The level of maturity of the pharmaceutical regulatory system is low (level 1 on a scale of 1 to 4), with a lack of autonomy and independence of the ANRP.

**Sample size and facilities selection:** convenience sampling was used in this study, which is a non-probability sampling technique where subjects are selected because of their convenient accessibility and proximity to the researchers. This study population consists of public health facilities (national hospitals, provincial hospitals, district hospitals, health centers), religious health facilities, public pharmaceutical establishments (pharmaceutical purchasing center, provincial supply pharmacies), and private pharmaceutical establishments (private wholesalers, pharmacies, and pharmaceutical depots).

In the selection of public sector, all five (5) national hospitals and the pharmaceutical purchasing center were selected for the survey; at the provincial level, the 23 provincial hospitals and the 23 provincial supply pharmacies were selected; at the health district level, a little over 30% of functional district hospitals were selected by including for each province, an urban district hospital (the district hospital of the provincial capital) and a randomly selected rural district hospital; then 5 health centers in the area of each district are randomly selected. This approach was consistent with the Health Action International (HAI) method for selecting health facilities at the district level. In the selection of private sector, the pharmacy, or where appropriate, the pharmaceutical depot closest to each selected district hospital is included in the sample; the 3 main private wholesalers in Ndjamena in terms of volumes of imports of medical products and the only private wholesaler established in a province other than Ndjamena were included in the survey.

**Data collection and management:** the computer databases of pharmaceutical wholesalers and provincial supply pharmacies, paper and computer dispensing registers of health facilities, and price labels at the pharmacy level constituted the source of data. A standardized data collection form was designed on the basis of the 64 essential medicines and used to ensure data accuracy and reliability. Information on availability and cost was obtained using an interviewer-administered questionnaire. Seven trained teams of investigators, composed mainly of pharmacists and pharmacy technicians, were formed. For each drug, the price and availability in the healthcare facility at the time of the investigators' visit are provided. Among the presentations of the available drugs, the lowest priced generics (LPGs) and originator brands (OBs) were listed. Each drug is identified by its international nonproprietary name (INN), pharmaceutical form, dosage, and packaging. For other medical products, including medical devices, the cheapest item is listed. Prices were converted from CFA to USD based on the rate during the period of data collection. The exchange rate of CFA to USD equivalent was considered by taking the monthly average of June to July, 2023 (1 CFA= 0.00165 USD). Data were double-entered, and the data checker function on the spreadsheet was used to avoid data entry errors. Availability was assessed based on the number of medical products available in the basket of 64 essential medicines on the day of the survey (Table 1 and Table 1.1). The price difference between each OBs and its LPGs equivalent and the price and availability ratios of medical products in the different private and public sub-sectors were included as well. International prices were obtained from HAI [10].

**Table 1:** medicines surveyed, strength, dosage, and national essential medicines

No	Name	Strength	Dosage form	NEM	Affordability analysis
1	Lysine acetylsalicylate	1000mg/10ml	inj	Yes	-
2	Albendazole	400 mg	tab	Yes	Yes
3	Allopurinol	100mg	tab	Yes	-
4	Aluminum Hydroxide	400 mg	tab	Yes	-
5	Amlodipine	5 mg	tab	Yes	-
6	Amoxicillin	250mg/5ml	Susp	Yes	Yes
7	Amoxicillin	500 mg	Caps	Yes	-
8	Ampicillin	1g	inj	Yes	-
9	Human tetanus antitoxin	1500 UI	inj	Yes	-
10	Artemether + lumefantrine 120mg	20mg/120mg	tab	Yes	Yes
11	Artesunate	60mg	inj	Yes	Yes
12	Benzathine benzylpenicillin	2.4 MIU	inj	Yes	-
13	Carbamazepine	200mg	tab	Yes	-
14	G22 Catheter	-	-	Yes	-
15	Ceftriaxone	1g	inj	Yes	Yes
16	Chlorpromazine	25mg/mL	inj	Yes	-
17	Cimetidine	200 mg/mL	inj	Yes	-
18	Ciprofloxacin	500mg	tab	Yes	-
19	Ciprofloxacin	2mg/ml	100ml flacon	Yes	-
20	Cloxacillin	500mg	tab	Yes	-
21	Cloxacillin	250mg	cap	Yes	-
22	Colchicin	1mg	tab	Yes	-
23	Cotrimoxazole	200/40/5ml	Susp	Yes	-
24	Cotrimoxazole	400/80mg	Caps	Yes	-
25	Dexamethasone	4mg/ml	inj	Yes	-
26	Diazepam	5 mg	tab	Yes	-
27	Diazepam	5mg/2ml	inj	Yes	-
28	Diclofenac	50 mg	tab	Yes	-
29	Diclofenac	25 mg/ml	inj	Yes	-
30	Doxycycline	100mg	cap	Yes	-
31	Distilled water	5 ml	inj	Yes	-
32	Enoxoparin	4000 UI	inj	Yes	Yes
33	Erythromycin	500 mg	tab	Yes	-
34	Iron sulfate + Folic acid	60mg+0,4mg	tab	Yes	Yes
35	Furosemide	20mg/2ml	inj	Yes	-
Cap: Capsule; INJ: Injection, NEM: National Essential Medicines; SUSP: Suspension; TAB: Tablet					

**Table 1.1:** medicines surveyed, strength, dosage, and national essential medicines

1	Gentamycin	80 mg/2ml	inj	Yes	-
2	Glibenclamide	5mg	tab	Yes	Yes
3	Calcium gluconate	10%	inj	Yes	-
4	Slow or rapid insulin	100 units/ml	inj	Yes	-
5	Magnesium sulfate	0,5g/ml, amp/2ml	inj	Yes	-
6	Mebendazole	100 mg	inj	Yes	-
7	Metformin	500 mg	tab	Yes	-
8	Methyldopa	250 mg	tab	Yes	-
9	Metoclopramide	5mg/ml	inj	Yes	-
10	Metronidazole	125mg/5ml	Susp	Yes	-
11	Metronidazole	250mg	Tab	Yes	-
12	Nystatin	100000 IU	Susp	Yes	-
13	Nystatin	100000 IU	Ovule	Yes	-
14	Oxytocin	5 mg	inj	Yes	Yes
15	Omeprazole	20 mg	tab	Yes	-
16	Paracetamol	2.4 mg/ml	susp	Yes	-
17	Paracetamol	500mg	tab	Yes	-
18	Infusor	-	-	Yes	-
19	Phenobarbital	100mg	tab	Yes	Yes
20	Phloroglucinol	10mg/ml	inj	Yes	-
21	Polyvidone iodine	10%	Solution	Yes	-
22	Quinine	300mg	inj	Yes	-
23	Quinine	300mg/ml	inj	Yes	-
24	Ringer lactate	500 ml	Solution	Yes	-
25	Salbutamol	5mg/ml	inj	Yes	Yes
26	Oral Rehydration Salts Powder/Zinc	-	tab	Yes	-
27	Isotonic Glucose Solution	5%	Solution	Yes	-
28	Isotonic saline solution	0.9%	Solution	Yes	-
29	Tetracycline	0.01g	eye ointment	Yes	-
CAP: Capsule; INJ: Injection, NEM: National Essential Medicines; SUSP: Suspension; TAB: Tablet					

### Outcomes measures

*Availability:* the indicator used represents the overall mean availability of each of the samples of medicine in the facilities surveyed.

*Affordability:* this indicator was calculated for those medicines available in three or more outlets per sector. The affordability was evaluated using days' earnings, which were calculated by multiplying the mean drug price discovered in facilities by the number of units in a regular course of treatment, and then dividing by the lowest-paid government worker's daily income [10]. According to Africa HR, the minimum wage in Chad is FCFA 303/hour for agricultural and associated industries, and FCFA 355/hour for all other sectors. This corresponds to $0.49/hour and $0.57/hour, respectively [11].

$$\text{Affordability}=\frac{The\;total\;mean\;price\;of\;the\;regimen\;for\;a\;given\;drug\left(USD\right)}{The\;daily\;wage\;of\;the\;lowest\;paid\;goverment\;employee\;\left(USD\right)}$$

*Pricing:* three types of price comparison were made as part of the medicines access survey to help understand price levels in Chad and their variation by type of medicine.

**Statistical analysis:** this study focused on three key endpoints: medicine availability, patient prices, and affordability. The availability of individual medicines was calculated as the percentage (%) of the surveyed outlets where the medicine was found on the day of data collection. The median price ratio (MPR) is the ratio of the local median unit price of a medicine divided by the median IRP, as described in the equation below. The MPRs were calculated to express how much greater or less the median local medicine price was than the international reference prices (IRP). After data collection, data were entered and analyzed using Excel® WHO/HAI Medicine Pricing Workbook, and the results were summarized and presented in tables and graphs. Medicine availability was calculated as the percent availability of individual medicines; the mean (average) percent (%) availability across a group of medicines. Where prices were collected as mean (SE), a t-test comparing two groups, including public vs. private/contractual sectors. P-value? 0.05 was considered statistically significant. This analysis was undertaken using Stata 18 MP.

$$MPR\;of\;a\;specific\;medicine=\frac{median\;local\;unit\;price}{international\;reference\;unit\;price}$$

**Ethical considerations:** to carry out this work, we obtained the authorization of the Secretary General of the Ministry of Health, that of the National Order of Pharmacists, and that of the managers of the different structures surveyed. an information note from the secretary-general was shared with the health facilities visited. Furthermore, the data collected were anonymous, and the names of the structures do not appear in the tools and results.

## Results

**Figure 1:**
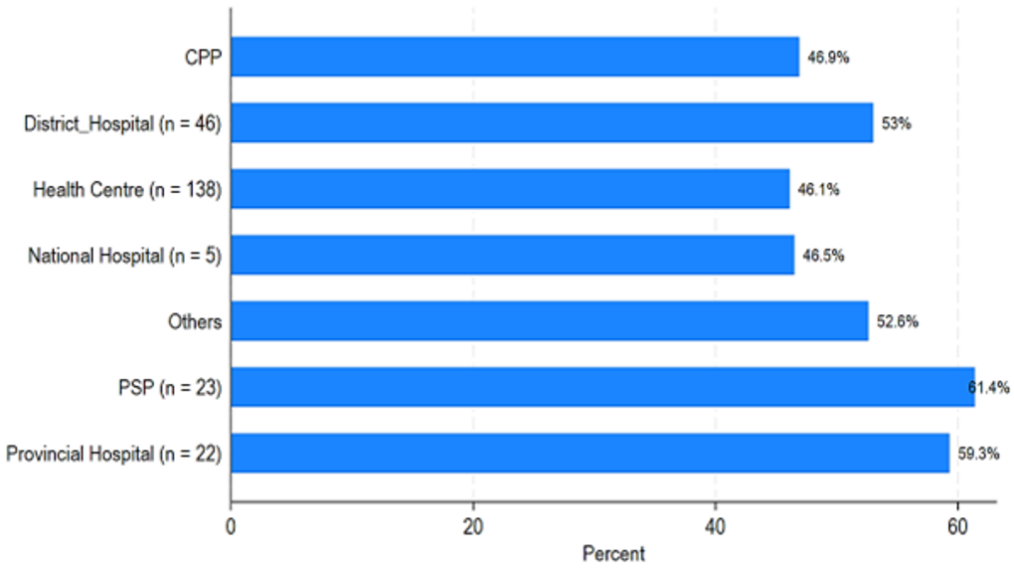
average availability of essential medical products in the public health sector, CPA: Central Pharmaceutical Purchasing Agency; PPA: Provincial Supply Pharmacy

**Figure 2:**
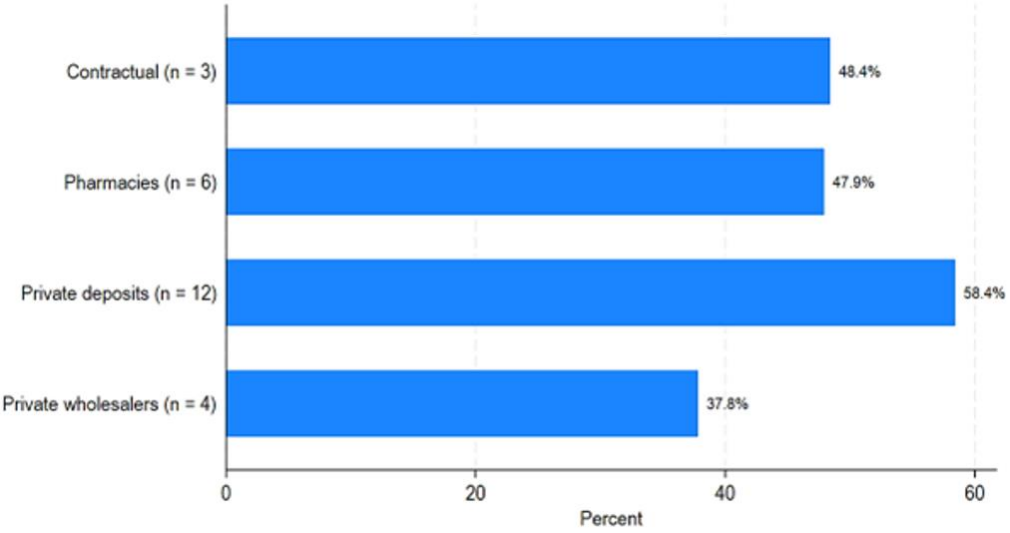
average availability of medical products in the private health sector and in approved or denominational health facilities

**Figure 3:**
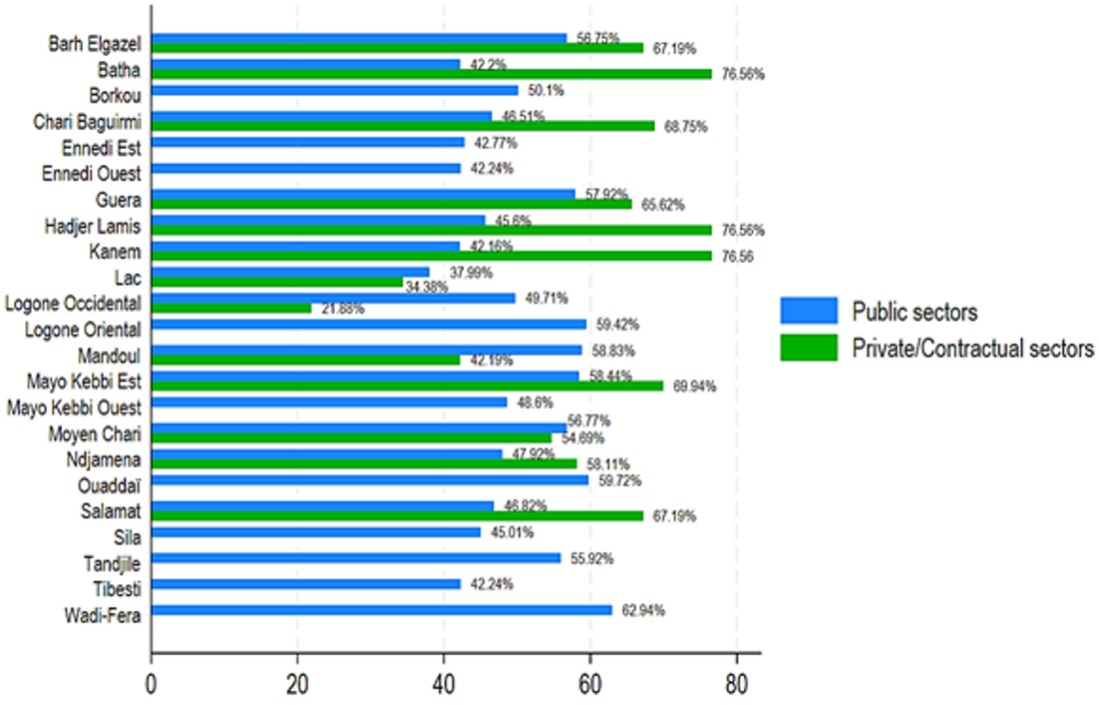
average medicine availability by province by public and private/contractual sectors

**Availability of essential medicines in Chad:** Figure 1 and Figure 2 show the availability of the 64 medical products as an average percentage of products available at the time of the surveyors' visits. In the public sector, the average availability varies between approximately 46% at the health center level and 61.4% at the provincial pharmacy level, with an average for all health facilities (hospitals and health centers) of 52.6% (Table 2 and Figure 1). At the private sector level, it is approximately 38% for private wholesalers, approximately 48% for pharmacies, and 58.4% for pharmaceutical depots. At the level of the contracted or denominational facilities, it is 48% (Table 2 and Figure 2). Figure 3 shows the average availability by province by public and private/contractual structures.

**Table 2:** medicine availability by sector

Sectors	Mean availability (%)
**Public**	
CPP	46.9
PSP (N= 23)	61.4
Average for all health facilities (Hospitals and health centers)	44.6
National hospitals (N = 5)	46.5
Provincial hospitals (N=22)	59.3
District hospitals (N=46)	53.0
Basic health centers (138)	38.8
**Private and conventional**	
Private wholesalers (n=4)	37.8
Pharmacies (n=6)	47.9
Private Deposits (n=12)	58.4
Contractual (n=3)	48.4
CPP: Central Pharmaceutical Purchasing; PSP: Provincial Supply Pharmacy	

**Figure 4:**
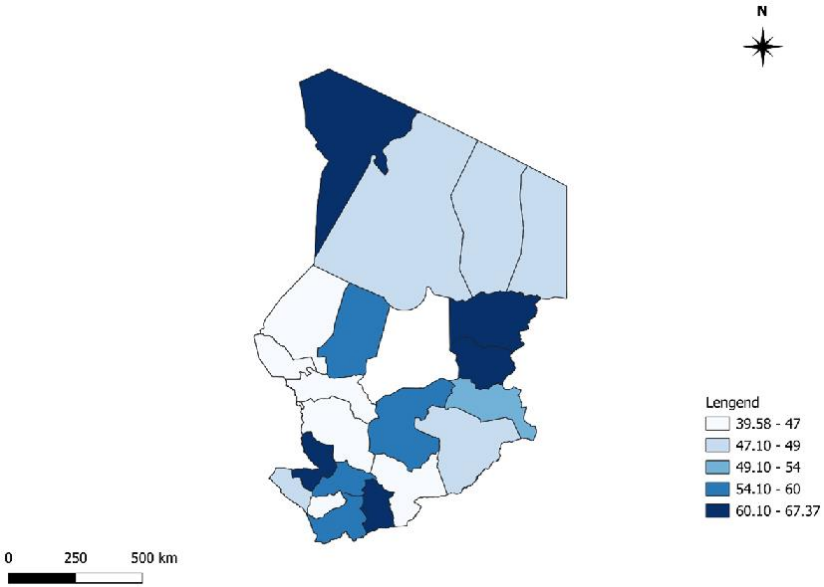
average essential medicine availability by province in Chad

Figure 3 shows the distribution of average essential medicine availability by province, by public and private/contractual structures. Our results showed that private/contractual vs. public showed higher availability with Ndjamena (58.12% vs. 47.92%), Massenya (68.75% vs. 46.51%), Mongo (65.62% vs. 57.92%), Moussoro (67.19% vs 56.75%), Ati (76.56% vs. 42.20%), Mao (76.56% vs. 42.16%), Massakory (76.56% vs. 45.6%), Bongor (69.94% vs. 58.44%). In contrast, private reported higher medicine availability than privates in Bol (37.99% vs. 34.38%), Sarh (56.77% vs. 54.69%), Moundou (49.71% vs. 21.88%), and Koumra (58.83% vs. 42.19%) (Table 3 and Figure 3). Figure 4 shows the average medicine availability by province in Chad from 1^st^ June to 31^st^ July 2023. This figure showed that the average medicine availability ranged between 39.59% and 67.37% (Figure 4). The provinces with the lowest availability were Batha, Kanem, Lac, and Hadjer Lamis (39% to 43%), whereas Tibesti, Mandoul, Wadi Fira, Mayo Kebi Est, Logone Oriental, and Ouaddaï have the most availability (60% to 67%) (Figure 4).

**Table 3:** medicine availability by province and health sector in Chad

Province	Public sector Mean%(SE)	Private sector (Mean%(SE)	t-test	P-value
Ndjamena	47.92(3.12)	58.12(11.62)	-4.37	0.0002
Chari Baguirmi	46.51(3.07)	68.75(19.85)	-3.88	0.0026
Guera	57.92(3.82)	65.62(18.94)	-4.72	≤0.0001
Salamat	46.82(3.09)	67.19(19.52)	-13.34	≤0.0001
Ouaddaï	59.72(3.94)	-	-	-
Sila	45.01(2.97)	-	-	-
Wadi-Fera	62.94(4.15)	-	-	-
Ennedi Est	42.77(2.82)	-	-	
Barh Elgazel	56.75(3.74)	67.19(19.40)	-6.37	≤0.0001
Batha	42.2(2.81)	76.56(22.10)	-5.38	0.0002
Kanem	42.16(2.80)	76.56(22.10)	-5.38	0.0002
Lac	37.99(2.50)	34.38(9.92)	1.26	0.2341
Hadjer Lamis	45.60(3.00)	76.56(22.10)	-4.85	0.0005
Mayo Kebbi Est	58.44(3.85)	69.94(20.19)	-2.78	0.0100
Mayo Kebbi Ouest	48.60(3.20)	-	-	-
Tandjile	55.92(3.69)	-	-	-
Moyen Chari	56.77(3.74)	54.69(15.79)	0.66	0.4557
Logone Occidental	49.71(3.28)	21.88(6.32)	15.14	≤0.0001
Logone Oriental	59.42(3.92)	-	-	-
Mandoul	58.83(3.88)	42.19(12.18)	4.72	0.0006
Borkou	50.10(3.30)	-	-	-
Ennedi Ouest	42.24(2.78)	-	-	-
Tibesti	42.24(2.78)	-	-	-

**Figure 5:**
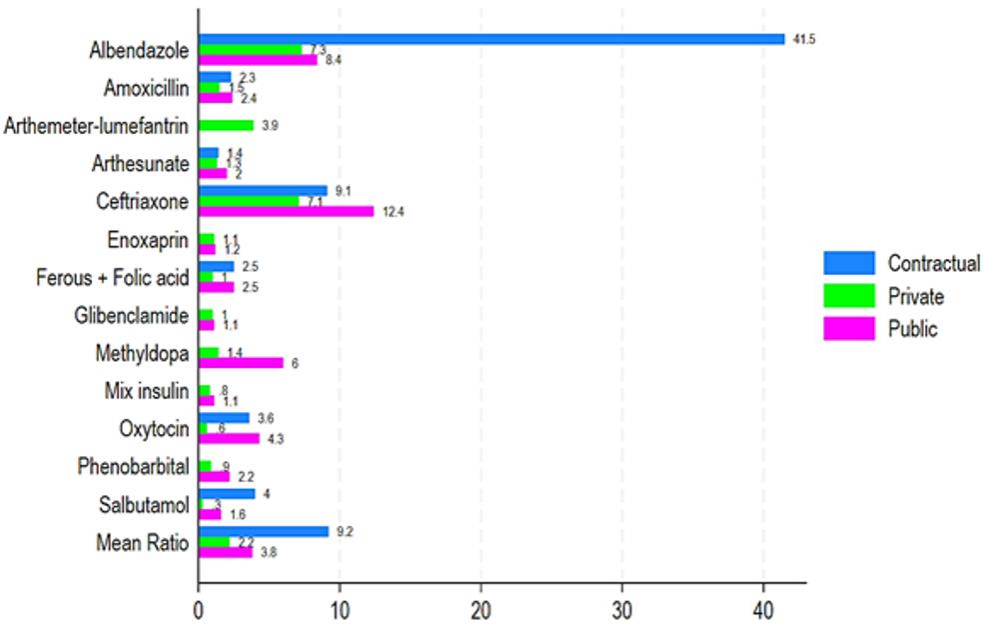
pricing ratios of medical items relative to the originator's pricing

**Price of essential medicines in Chad:** Figure 4 shows the MPR of the average price (Princeps/Public; Princeps/Private; Princeps/contracted) of some medical products compared to the price of the Princeps available on the market (pharmacy). The MPR revealed that the cheapest generic costs 9 times less than the princeps in contracted/denominational health facilities, approximately 4 times less in the public sector, and roughly 2 times less in the private sector (Figure 5). The MPR of albendazole was higher in contractual structures (41.5) compared to private (7.3) and public (8.4) (Figure 5). Slight variations on MPR of amoxicillin were found in contractual and private sectors (2.3 vs. 2.4) and were lower in private (1.5) (Figure 5). The MPR of artesunate was 1.4, 1.3, and 2 for contractual, private, and public structures, respectively (Figure 5). Higher MPR for ceftriaxone was observed in private (12.4) compared to contractual (9.1) and public structures (7.3). About enoxaparin, a slight difference was observed between the private (1.1) and public sectors (1.2) (Figure 5). Contractual and public sectors of MPRs were similar (2.5), compared to a lower MPR in private (1), and glibenclamide had quite similar MPR in both and private and public, roughly 1 (Figure 5). Methyldopa and mixed insulin had higher MPRs in private vs. public sectors with 6 vs. 1.4 and 1.1 vs. 0.8, respectively (Figure 4). MPR of oxytocin was higher in contractual (3.6) and private (4.3), compared to the public sector (0.6) (Figure 5). Phenobarbital had a lower MPR in the private (0.6) than in the public sector (2.2). Lastly, salbutamol had a higher MPR in contractual structures (4) compared to public (0.3) and private sectors (1.6) (Figure 5).

**Affordability of essential medicines in Chad:** Table 4 shows the affordability of essential medicines in Chad. Our results showed that the mean price of all of the medicines was found to be unaffordable, costing more than one day's wage in both the public and private/contractual sectors (Table 4). All the means for artesunate, amoxicillin, artemether-lumefantrine, ceftriaxone, iron sulfate + folic acid, glibenclamide, mixed insulin, methyldopa, oxytocin, enoxaparin, phenobarbital, and salbutamol were statistically affordable in the public sector than in the private/contractual sector (Table 4). The result of the overall affordability calculation revealed that the top three unaffordable medicines included enoxaparin, ceftriaxone, and mixed insulin with 246.14, 56.12, and 32.35 days' wages of the lowest-paid government worker, respectively.

**Table 4:** affordability of essential medicines (USD) by public and private/contractual sectors

Medicine	Strength	Dosage form	Regimen for a given drug	Public (n = 30)	Private/Contractual (n = 32)	t-test	P-value
Albendazole	400 mg	tablet	Box of 1	1.66(0.23)	1.90(0.24)	-4.01	<0.001
Artesunate	100 mg	tablet	Box of 28	2.34(0.28)	4.41(0.37)	-24.71	<0.001
Amoxicillin	250mg/5ml	susp	60 mL bottle	2.77(0.30)	4.36(0.37)	-18.51	<0.001
Artemether -Lumefantrin	20/120 mg	tablet	Box of 24	0(0.00)	3.94(0,35)	-61.62	<0.001
Ceftriaxone	1g	inj	Seven Powder Bottle	15.87(0.73)	27.50(0.93)	-54.52	<0.001
Enoxaprin	4000 UI	inj	Box of 10	120.61(2.00)	127.09(1.99)	-12.78	<0.001
Iron sulfate + Folic acid	60mg+0,4mg	tab	Box of 100	6.58(0.46)	17.11(0.73)	-67.43	<0.001
Glibenclamide	5 mg	tab	Box of 20	5.47(0.43)	6.22(0.44)	-6.78	<0.001
Mixed Insulin	Prefilled Syringe	inj	Box of 1	15.85(0.73)	22.41(0.84)	-32.73	<0.001
Methyldopa	250 mg	tab	Box of 30	3.90(0.36)	16.34(0.71)	-86.12	<0.001
Oxytocin	5 mg	inj	Box of 1	1.07(0.19)	7.26(0.48)	-65.93	<0.001
Phenobarbital	100mg	tab	Box of 30	5.26(0.42)	12.38(0.62)	-52.58	<0.001
Salbutamol	5mg/ml	inj	One ampoule	0.74(0.16)	4.66(0.38)	-52.30	<0.001
CAP: Capsule; INJ: Injection; NEM: National Essential Medicines; SUSP: Suspension; TAB: Tablet							

**Figure 6:**
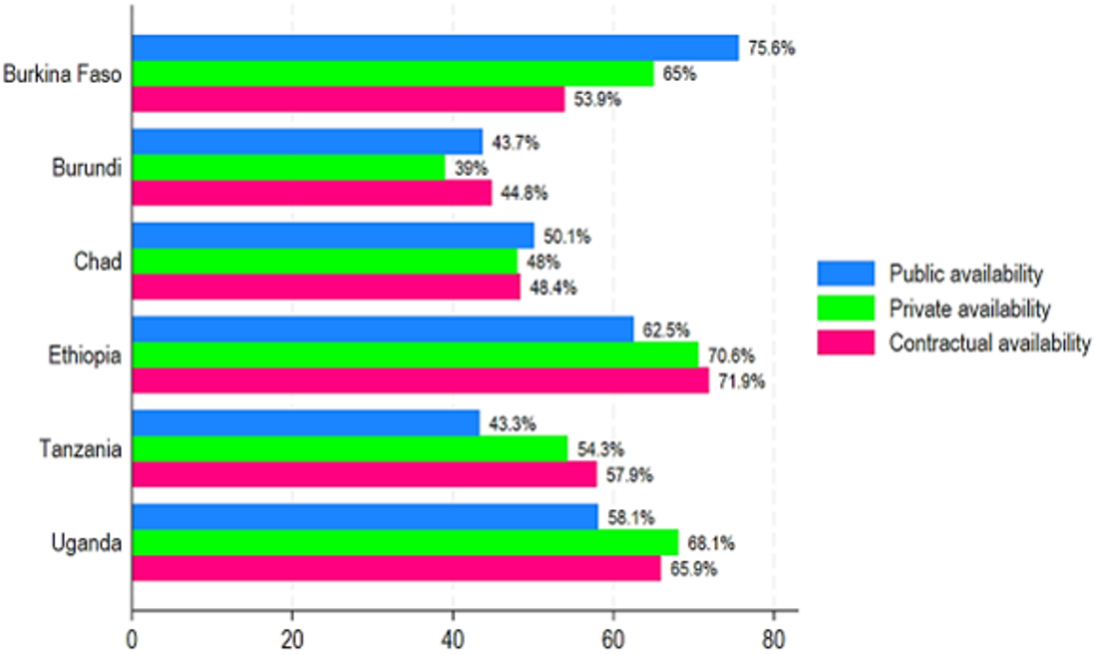
availability (%) of essential medicines by public, private, and contractual sectors in Chad compared to other sub-Saharan African countries

**Figure 7:**
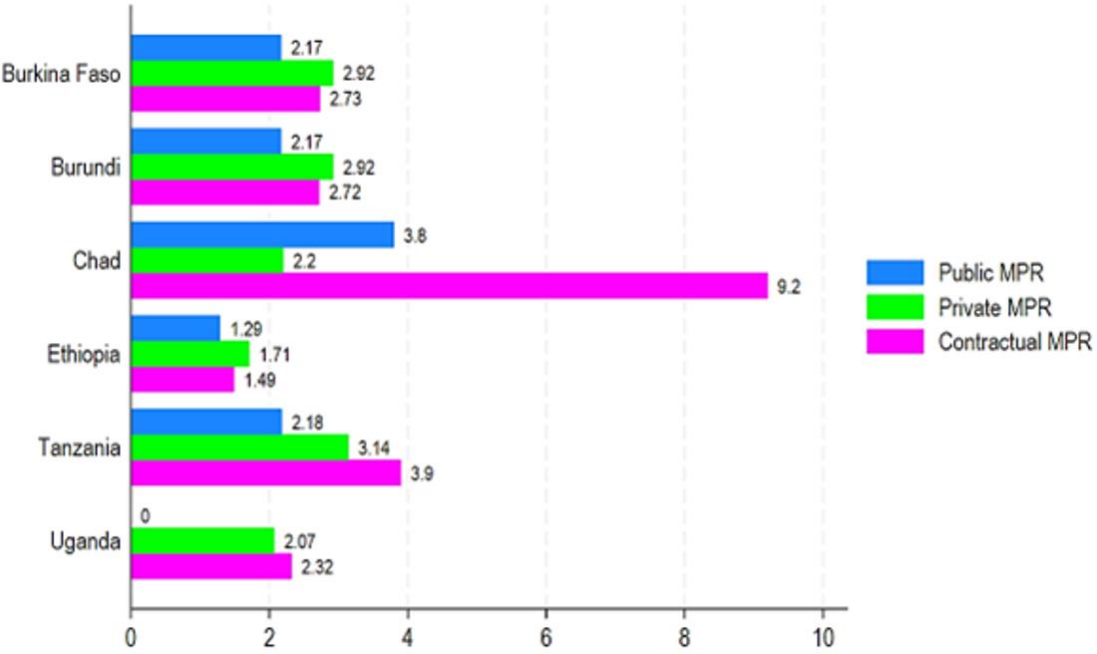
affordability of the lowest price by public, private, and contractual sectors in Chad compared to other sub-Saharan African countries

**Medicines availability and pricing in Chad compared to other sub-Saharan African countries:** in comparison to five sub-Saharan African countries that have published their surveys on WHO/HAI website, Chad had higher medicine availability compared to Burundi in all three sectors, including public, private, and contractual. Furthermore, Chad had slightly higher essential medicines availability in public sectors compared to Tanzania (50.1% vs. 43.3%) (Figure 6). Besides, Chad had lower essential availability compared to Burkina Faso, Ethiopia, and Uganda (Figure 6). Regarding essential medicines affordability, Chad had the highest MPRs in public and contractual sectors compared to Burkina Faso, Burundi, Ethiopia, Tanzania, and Uganda (Figure 7). When we compared Chad's pricing to that of other sub-Saharan countries, we found that LPGs were around six times more expensive than the lowest price in Ethiopia's contractual sector (Figure 7). In the public sector, LPGs were nearly three times more expensive in Chad than in Ethiopia's lowest pricing (Figure 7).

## Discussion

We did a study from 1^st^ June to 31^st^ July 2023, in Chad. The study looked at public, private, and other sectors to find out how available essential medicines are. We checked how much patients pay for these medicines, looked at the price difference between brand-name drugs and the cheapest generic versions, and compared prices and availability of medicines in different sectors. We also compared prices in Chad to those in other sub-Saharan African countries. The availability of necessary medicines varied greatly in different provinces of Chad. Our results showed that the average supply of medicine was between 39.58%% and 67.37%. The most impacted provinces, such as Batha, Lake, Hadjer Lamis, and Kanem, have extremely low availability of essential medicines, which can substantially jeopardize disease management in these areas. The observed differences between provinces can be attributed to complex factors, such as local health infrastructure, distribution policies, and allocated health budgets, highlighting the need for targeted interventions to address these disparities [12,13]. There was more essential medicine availability in the private or contract sectors than in the state sector. The overall availability of 52.6% in the public sector, as well as similar figures in the private sector (58.4% in depots and 48% in pharmacies), highlights serious gaps in access to treatment in Chad. This observation is part of an African context where the availability of essential medicines often remains insufficient, directly impacting health outcomes [14]. Our results were in line with a recent study that has shown that the average availability of generic medicines across WHO regions ranged from 37.8% to 68.3% in the public sector and from 42.3% to 77.4% in the private sector. The availability of originator brand medicines in the private sector ranged from 18.0% to 47.6% across these regions. Neither the public nor the private sector in any region met WHO's recommended availability target of 80% [15]. The study highlights a worrying situation regarding the availability and prices of essential medicines in Chad. In the absence of such access to medicines, whether due to unaffordability, unavailability, or poor quality, a violation of this right occurs [15].

In Chad, the MPR showed that the lowest-priced generic medicine is 9 times cheaper than the brand-name version in leased health facilities. In the public sector, it is about 4 times cheaper, and in the private sector, it's roughly 2 times cheaper. Among thirteen essential medicines assessed for affordability, eleven were more affordable to the public sector than the private/contractual sectors. Medicine prices were consistently high across all WHO regions, requiring patients to pay 3.0-11.5 times international reference prices for LPGs medicines and over 25 times international reference prices for OBs across WHO regions [15]. Price remains a major factor of inefficiency in health systems in Africa, where patients must face high costs, which can limit access to treatments and worsen health inequalities [16]. The price difference, even in the private sector, shows that substantial savings could be made if patients opted for generic alternatives, but this depends on their availability. A study carried out in Kenya showed that generic medicines were on average 3.5 times cheaper than their originator equivalents in the public sector [17]. These results are consistent with our study, but the more pronounced gap in the conventional sector in Chad suggests an urgent need for public health policies aimed at improving access to generic medicines and strengthening regulation of the pharmaceutical market. It is well documented that the use of generics, although often more economical, is not systematically adopted due to perceptions about their effectiveness and quality [18]. Awareness-raising policies and educational campaigns can be essential to encourage the use of generics and reduce inequalities in access to care.

In comparison to five SSA countries that have published their surveys on WHO/HAI website, Chad had higher medicine availability compared to Burundi in all three sectors, including public, private, and contractual. Furthermore, Chad had slightly higher essential medicines availability in the public sector compared to Tanzania. Besides, Chad had lower essential availability compared to Burkina Faso, Ethiopia, and Uganda. Regarding essential medicines affordability, we found that LPGs were around six times more expensive than the lowest price in Ethiopia's contractual sector. In the public sector, LPGs were nearly three times more expensive in Chad than the lowest price in Ethiopia. A study revealed that the comparison of pricing variances by medicine between health institutions demonstrates that the order on price harmonization is not fully implemented in Chad [7]. This scenario is due in part to insufficient oversight and a shortage of pharmaceutical inspection resources in Chad [7]. Studies on access to care in Africa, particularly those carried out in Tanzania and Zambia, have also shown marked regional disparities in essential medicines availability and affordability, linked to socio-economic factors, stock management, and health infrastructure [19,20]. Availability and affordability are considered the key requisites for universal access to medicine [21]. When compared with the public, private, and contractual sectors, the availability of essential medicines is still below the WHO touchstone of 80% regarding availability in Chad. To address the poor availability of both public and private/contractual sectors, as well as in all the provinces in Chad, more specific guidance for the preferential use of essential medicines should be issued by the government to improve people´s access to them. There was evidence that the implementation of medicine pricing policy led to improved availability and affordability of essential medicines [22]. Adequate financing to pay for an appropriate set of essential medicines is the first key challenge in Chad. Medicines represent a large proportion of household expenditure on health in LMICs such as Chad [23].

According to the World Health Survey, up to 9.5% of the total expenditure of poorer households in LMICs is spent on medicines, far higher than the 3.5% expended by poorer households in high-income countries (HICs) [24]. This statistic is particularly true in countries where inadequate public financing of health care results in high out-of-pocket expenditure [25]. Understanding medicine pricing policy implementation is particularly important as implementation of these policies can be a major challenge in LMICs where many patients with extremely limited resources need to provide out-of-pocket payments, thus impeding their access to medicines and putting them at further risk with increasing prices [22,26,27]. WHO has formulated a four-part framework to guide and coordinate collective action on access to essential medicines [28]. This framework, adopted by WHO's key partners, comprises rational selection, affordable prices, sustainable financing, and reliable health and supply systems. By scaling up existing interventions for infectious diseases, maternal and child health, and noncommunicable diseases. Most of these interventions depend on essential medicines [28]. According to the WHO report on universal health coverage, approximately 4.5 billion people worldwide do not have access to essential health services, including medicines [29].

This study has several limitations, including the fact that several health facilities, including health centers, were difficult to access due to the rainy season, which made access roads impassable. However, this limitation did not affect the general trend of the results, which cover all provinces of the country. Studies on the challenges of access to care in sub-Saharan Africa highlight that transport infrastructure and climatic conditions play an important role in the availability of care in rural areas [30]. Despite these challenges, the data collected covers a large part of the country and broadly reflects the health situation in Chad. A major strength of this study is the use of the WHO/HAI medicine survey, which allowed us to measure availability, prices, and affordability in a reliable and standardised way. This study's findings can be used to design public policies targeted at enhancing access to medicines by informing decision-makers about current challenges.

## Conclusion

The findings of this study emphasize the vital need for essential medicines and for lowering their costs in Chad. Efforts should be made to apply the WHO recommendations for universal access to essential medicines in Chad. Initiatives to build health infrastructure, promote the availability of generic drugs, and expand health coverage are required. Strict regulatory measures, notably for managing drug costs and monitoring distribution methods, are required to increase access and quality of care. These steps are critical not only for reducing health disparities but also for promoting the country's economic development, as affordable health care drives growth. Furthermore, the distribution of the results will help to raise awareness among health sector stakeholders, especially non-governmental organizations (NGOs) and donors, about the current state of medications in Chad.

### What is known about this topic

- Chad has a weak health system with limited access to medical care for the population;
- Elevated prices, combined with diminished purchasing power, restrict the financial accessibility of most individuals to pharmaceuticals in Chad;
- The last assessment of the availability and price of essential medicines in Chad took place in 2004.

### What this study adds

- In Chad, the availability of necessary medicines differed significantly across provinces, private or contract sectors, and the governmental sector;
- In Chad, the median price ratio (MPR) showed that the lowest-priced generic medicine is 9 times cheaper than the brand-name version in leased health facilities;
- In sub-Saharan Africa, the lowest priced generics (LPGs) were around six times more expensive in Chad than the lowest price in Ethiopia's contractual sector.
